# SRC-1 Regulates Blood Pressure and Aortic Stiffness in Female Mice

**DOI:** 10.1371/journal.pone.0168644

**Published:** 2016-12-22

**Authors:** Antentor Othrell Hinton, Yongjie Yang, Ann P. Quick, Pingwen Xu, Chitra L. Reddy, Xiaofeng Yan, Corey L. Reynolds, Qingchun Tong, Liangru Zhu, Jianming Xu, Xander H. T. Wehrens, Yong Xu, Anilkumar K. Reddy

**Affiliations:** 1 Pediatrics-Children’s Nutrition Research Center, Baylor College of Medicine, Houston, Texas, United States of America; 2 Department of Molecular Physiology and Biophysics, Baylor College of Medicine, Houston, Texas, United States of America; 3 Cardiovascular Research Institute, Baylor College of Medicine, Houston, Texas, United States of America; 4 Debakey High School for Health Professions, Houston, Texas, United States of America; 5 Advanced Technology/Core Laboratory, Baylor College of Medicine, Houston, Texas, United States of America; 6 Brown Foundation Institute of Molecular Medicine, University of Texas Health Science Center at Houston, Houston, Texas, United States of America; 7 Department of Gastroenterology, Union Hospital, Tongji Medical College and Huazhong University of Science and Technology, Wuhan, China; 8 Department of Molecular and Cellular Biology, Baylor College of Medicine, Houston, Texas, United States of America; 9 Section of Cardiovascular Research, Department Medicine and DeBakey Heart Center, Baylor College of Medicine, Houston, Texas, United States of America; 10 Indus Instruments, Webster, Texas, United States of America; Seconda Universita degli Studi di Napoli, ITALY

## Abstract

Framingham Heart Study suggests that dysfunction of steroid receptor coactivator-1 may be involved in the development of hypertension. However, there is no functional evidence linking steroid receptor coactivator-1 to the regulation of blood pressure. We used immunohistochemistry to map the expression of steroid receptor coactivator-1 protein in mouse brain, especially in regions implicated in the regulation of blood pressure. Steroid receptor coactivator-1 protein was found in central amygdala, medial amygdala, supraoptic nucleus, arcuate nucleus, ventromedial, dorsomedial, paraventricular hypothalamus, and nucleus of the solitary tract. To determine the effects of steroid receptor coactivator-1 protein on cardiovascular system we measured blood pressures, blood flow velocities, echocardiographic parameters, and aortic input impedance in female steroid receptor coactivator-1 knockout mice and their wild type littermates. Steroid receptor coactivator-1 knockout mice had higher blood pressures and increased aortic stiffness when compared to female wild type littermates. Additionally, the hearts of steroid receptor coactivator-1 knockout mice seem to consume higher energy as evidenced by increased impedance and higher heart rate pressure product when compared to female wild type littermates. Our results demonstrate that steroid receptor coactivator-1 may be functionally involved in the regulation of blood pressure and aortic stiffness through the regulation of sympathetic activation in various neuronal populations.

## Introduction

Cardiovascular disease is the leading cause of morbidity as evidenced by 17.5 million reported deaths in 2012, of which women make up the majority of the 9 million patients that succumbed to hypertension [1]. It is estimated that people living with high blood pressure (BP) in the developing countries will increase from over 600 million at present to over 1.5 billion by 2025 [2]. Despite advances in treatment, hypertension continues to be poorly controlled as indicated by its increase over the past two decades. Hypertension is classified as either primary or secondary hypertension. Primary hypertension, also known as essential hypertension, is a disease of unknown etiology and constitutes about 95% of all cases [3]. Therefore, a better understanding of the causes of essential hypertension and novel therapies to treat and prevent it are urgently needed [3].

The female sex hormone, 17β-estradiol, has long been known to prevent development of hypertension. Pre-menopausal women have lower risks of hypertension than age-matched men [4]. However, after menopause the incidence of hypertension in women increases dramatically due to increase in BP caused by the drop in 17β-estradiol levels [5] and increase in aortic stiffness [6]. In various animal models of hypertension it has been shown that depletion of endogenous 17β-estradiol with ovariectomy exacerbated the course of hypertension, while 17β-estradiol replacement diminished it [7,8,9,10,11,12,13]. More recently, we showed that 17β-estradiol replacement significantly reduced stress-induced BP elevations in ovariectomized female mice [14]. Multiple clinical trials have also demonstrated the anti-hypertensive benefits of 17β-estradiol in post-menopausal women [15,16,17,18]. But, current estrogen replacement therapy is often associated with side effects, including enhanced risk of breast cancer [19]. Understanding the specific mechanisms for estrogenic actions to regulate BP may facilitate the development of novel estrogen-based therapies that provide much needed cardiovascular benefits with fewer side effects.

Multiple estrogen receptors are involved in anti-hypertensive effects of 17β-estradiol. For example, deletion of estrogen receptor-α (ERα) in mice blocks the vasoprotective effects of 17β-estradiol in angiotensin II-induced [11] and stress-induced [14] pressor responses. The ERα gene variants have been associated with hypertension in humans [20,21,22,23] and the estrogen receptor-β (ERβ) was shown to be associated with increase in BP in ERβ knockout mice [24]. The putative estrogen receptor, GPR30, is also involved in the regulation of BP, as deletion of this receptor leads to hypertension in mice [25]. While receptors that mediate estrogenic actions on BP have been reported, the molecular mechanisms by which 17β-estradiol regulates BP remain to be fully understood.

ERs act as classic nuclear receptors which induce gene transcription through association with nuclear receptor coactivators, including Steroid Receptor Coactivator-1 (SRC-1). SRC-1 is a member of the steroid receptor coactivator family that interacts with ERs to assemble a stable pre-initiation complex [26]. We previously showed that central administration of ERα agonist significantly enhances the interaction of SRC-1 and ERα in mouse hypothalamus [27]. This indicates that SRC-1 interacts with ERα in a ligand-dependent manner suggesting that SRC-1 may be required to mediate physiological functions of 17β-estradiol, such as the regulation of BP. Evidence from Framingham Heart human genetic study suggests that a single nucleotide polymorphism in the SRC-1 gene (rs1550383) is associated with significantly increased diastolic blood pressure in women but not in men [20]. These findings suggest that SRC-1 might contribute to the regulation of BP in a sexually dimorphic fashion. However, no biological evidence exists to support the possible role of SRC-1 in the regulation of BP. In this study we first examined SRC-1 protein distribution in mouse brain regions that are implicated in the regulation of BP. We then used a global SRC-1 knockout mouse model (SRC-1-KO) to assess if SRC-1 contributes to the regulation of BP, aortic stiffness, and overall cardiovascular function in female mice.

## Methods

### Animals

All the mice (2–4 mo. of age) used in this study were housed in a 12-h light and 12-h dark cycle. Mice were fed *ad libitum* normal chow diet (#2916, Harlan-Teklad, Madison, WI). Care of all the animals and the procedures used conformed to the Guide for Care and Use of Laboratory Animals of the US National Institutes of Health and were approved by the Institutional Animal Care and Use Committee of Baylor College of Medicine.

### SRC-1-KO mice

SRC-1-KO mice were generated from male and female heterozygous SRC-1^–/+^ crosses. These crosses generated litters containing homozygous SRC-1^–/–^(SRC-1-KO) and SRC-1^+/+^ (wild type, WT) littermates. The genotype of the mice was confirmed using tail DNA, qPCR and immunohistochemistry, as previously described [26,27,28].

### Immunofluorescence

Female mice were anesthetized with isoflurane during the light (inactive) period and were transcardially perfused with 60 ml of 0.9% saline and 200 ml of 10% formalin. Subsequently, the brain was removed and post-fixed for 3-hours in 20% sucrose and in 10% formalin. It was then placed in 30% sucrose in 0.1 M phosphate buffer pH 7.2 (PBS) and refrigerated overnight at 4°C. Coronal sections (25μm) were cut with Thermo Scientific HM 450 Sliding Microtome and placed in PBS. Free-floating sections were washed 6 times in PBS and incubated in 10% normal goat serum at room temperature for 2 hours. Next, the sections were incubated in the primary rabbit anti-SRC-1 serum (1:200; catalog no. sc-8995; Santa Cruz Biotechnology) at room temperature overnight, followed by secondary Alexa-488 (Invitrogen) for 2 hours. Again, the free-floating sections were washed 3 times and mounted. Images were analyzed using a Leica 5500 fluorescence microscope (Leica, Heidelberg, Germany).

### DAB-immunohistochemistry

Brain sections were prepared as described above. The free-floating sections were washed 6 times in PBS and then incubated in 10% normal goat serum at room temperature for 2 hours. The sections were then incubated in the primary rabbit anti-SRC-1 serum (1:2000; catalog no. 2191, Cell Signaling, Beverly, MA) at room temperature overnight, followed by biotinylated anti-rabbit secondary antibody (1:1000; Vector Laboratories, Burlingame, CA) for 2 hours. Next, the sections were incubated in the avidin-biotin complex (Vector Elite kit; 1:500) and then in 0.04% diaminobenzidine and 0.01% hydrogen peroxide. After dehydration through graded ethanol, the slides were immersed in xylene and coverslipped. Images were analyzed using a Leica 5500 microscope configured with a bright-field camera (Leica, Heidelberg, Germany).

### Blood pressure measurements

Pressure measurements were made as previously described [29,30]. Briefly, mice were anesthetized in an induction chamber using 2.5% isoflurane and transferred to a heated electrocardiography board (MouseMonitor S, Indus Instruments, Webster, TX) with anesthesia maintained at 1.5% isoflurane. The paws of the mouse were taped to the four electrodes, the quality of electrocardiogram (ECG) assessed, and the electrode contacts optimized as needed. The neck area was shaved and using blunt dissection the right carotid artery of the mouse was isolated. The distal end of the carotid artery was tied off and its proximal end was temporarily closed. A small cut was made in the carotid artery and a 1F Millar catheter (SPR1000, Millar Instruments, Inc., Houston, TX) was inserted and secured with a suture tied over the artery-catheter overlap region. The proximal end was then opened and the catheter was advanced into the ascending aorta. Blood pressure and ECG signals were acquired using Doppler Flow Velocity System (DFVS; Indus Instruments, Webster, TX) and stored for analysis offline. The catheter was then advanced into the left ventricle to measure the left ventricular pressure. Systolic (SBP), diastolic (DBP), mean (MBP), and pulse pressure were extracted from aortic pressure signal. Peak left ventricular pressure (P_LVP_), maximal rate of change of contraction (+dP/dt_max_), maximal rate of change of relaxation (-dP/dt_max_), time constant of relaxation (tau), left ventricular end diastolic pressure (LVEDP) were extracted from left ventricular pressure signal.

### Echocardiography measurements

Cardiac dimensions and parameters were measured with a 30 MHz ultrasound echocardiography system (VEVO 770, Visualsonics, Toronto, Canada) to study *in-vivo* cardiac structure and function. Mice were anesthetized in an induction chamber using 2.5% isoflurane and then transferred to a heated electrocardiography platform for heart rate monitoring during the imaging procedure. The body temperature was monitored via rectal probe and was maintained at 37°C. Anesthesia was maintained via a nose cone at 1% isoflurane during imaging. Standard B-mode (2D) and M-mode images were taken in the short axis position at the level of the papillary muscles for each animal. Also, B-mode images of aortic arch were taken to measure aortic arch diameter. Data analysis was performed using the VEVO 770 Cardiac analysis package. Two M-mode images were analyzed per animal and averaged.

### Doppler flow velocity measurements

Blood flow velocity signals were measured in the anesthetized mice using a 20-MHz Doppler probe. Cardiac blood velocity signals were measured in the ascending aorta (aortic outflow) and at the mitral orifice (mitral inflow). The Doppler probe was positioned such that the angle between the sound beam and the direction of blood flow was less than 15° and the range gate depth was adjusted between 4–7 mm to obtain maximal velocity. Blood flow velocity signals were also measured at aortic arch and at abdominal aorta with separation distances between the two sites ranging from 3.5–4.1cm. The separation distance and transit time of flow velocity pulse was used to determine the aortic pulse wave velocity. Doppler aortic blood flow velocity and ECG signals were acquired using DFVS and stored for analysis offline [31]. Peak flow velocity was extracted from aortic blood flow velocity signal. Peak early (E) and atrial (A) velocities were extracted from mitral signal and peak E/A velocity ratio was calculated. Aortic pulse wave velocity (PWV_ff_) was calculated by dividing the separation distance by the transit time of the foot of the velocity waveforms from aortic arch to abdominal aorta (foot-to-foot method) [30,31].

### Determination of aortic input impedance

Aortic input impedance was determined using the simultaneously measured ascending aortic pressure and velocity signals. Care was taken to ensure that the Doppler sample volume was placed very close to the location of the pressure sensor such that the foot of the velocity signal was aligned with the foot of the pressure signal to minimize errors in phase relation between pressure and velocity signals [32]. Typically, 2–3 second segments of pressure, blood velocity, and ECG signals were acquired using DFVS and stored for offline analysis. The pressure and velocity waveforms were processed using discrete Fourier transform and aortic input impedance (Z_i_) was calculated [29,30]. Z_i_ was multiplied with the cross-sectional area (A) of aortic lumen to express it as volume flow based impedance. Peripheral vascular resistance (*Z*_P_), characteristic impedance (*Z*_C_), impedance at first harmonic (Z_1_) were extracted from modulus of aortic impedance (|Z_i_|). Impedance based pulse wave velocity, PWV_Z_ (= Z_C_•A/ρ; A—cross-sectional area of aortic lumen, ρ - density of blood) was calculated.

### Statistical analyses

All the data are presented as mean±SEM. Statistical analyses were performed using Prism (GraphPad Software). Group comparisons were performed using Student’s t-test with p<0.05 as the level of significance.

## Results

### Validation of SRC-1-KO mice

**Fig 1:**
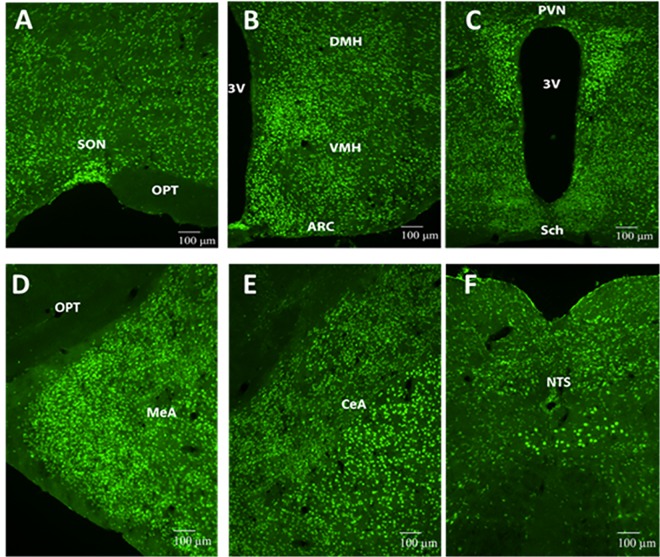
Expression of SRC-1 in the brain. Immunofluorescence staining of SRC-1 in various brain regions of WT female mice. 3V, third ventricle; ARC, arcuate nucleus of the hypothalamus; CeA, central amygdala; DMH, dorsal medial nucleus of the hypothalamus; MeA, medial amygdala; NTS, the nucleus of the solitary tract; OPT, optic tract; PVN, paraventricular nucleus of the hypothalamus; Sch, suprachiasmatic nucleus; SON, supraoptic nucleus; VMH, ventromedial nucleus of the hypothalamus (VMH). Scale bars = 100 μm.

**Fig 2:**
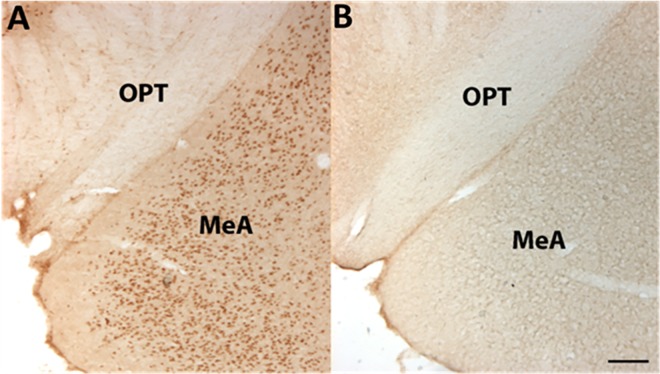
Validation of SRC-1-KO mice. 3,3′-diaminobenzidine immunohistochemistry staining for SRC-1 in the medial amygdala of female WT (**A**) or SRC-1-KO (**B**) mice. OPT, optic tract; MeA, medial amygdala. Scale bar = 100 μm.

We stained for SRC-1 in WT mouse brains using immunofluorescence to determine if SRC-1 co-localized to neuronal regions known to centrally regulate blood pressure. Analysis showed abundant SRC-1 immunoreactivity in the supraoptic nucleus (SON), arcuate nucleus (ARC), ventromedial (VMH), dorsomedial (DMH), paraventricular hypothalamus (PVN), medial amygdala (MeA), central amygdala (CeA), and nucleus of the solitary tract (NTS) (Fig 1A–1F). DAB-immunohistochemistry revealed that female WT mice had detectable protein levels of SRC-1 in the MeA (Fig 2A) while female SRC-1-KO mice had no detectable protein levels of SRC-1 in either the MeA (Fig 2B) or other brain regions (data not shown). SRC-1 KO demonstrated no staining thereby validating the successful deletion of SRC-1 in brain regions that may be important for the regulation of blood pressure.

### Blood pressure measurements

**Fig 3:**
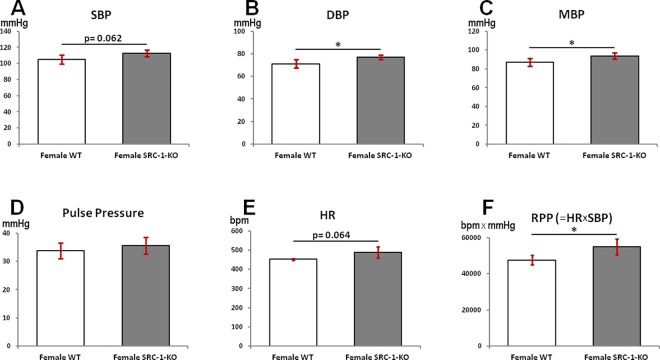
Aortic blood pressure and rate pressure product. Aortic blood pressure parameters of female WT and SRC-1-KO mice. (**A**) Systolic blood pressure (SBP), (**B**) Diastolic blood pressure (DBP), (**C**) Mean blood pressure (MBP), (**D**) Pulse pressure, (**E**) Heart rate, and (**F**) Rate pressure product (RPP). Data are presented as mean±SEM (n = 5-7/group); *—p*<* 0.05. The information supporting this figure is in S1 Dataset.

Aortic blood pressure was measured to determine the effect of SRC-1 deletion. Systolic (SBP), diastolic (DBP), mean (MBP), and pulse pressure were extracted from aortic pressure waveforms in female SRC-1-KO mice and female WT littermates. We found that the gonad-intact female SRC-1-KO mice had slightly higher SBP (p = 0.062, Fig 3A), significantly higher DBP (Fig 3B) and MBP (Fig 3C) when compared to female WT littermates. No significant differences were observed in the pulse pressure or the heart rate between the groups (Fig 3D and 3E). However, rate pressure product (SBP x heart rate) was significantly higher in the female SRC-1-KO mice when compared to female WT littermates (Fig 3F, p = 0.026).

**Fig 4:**
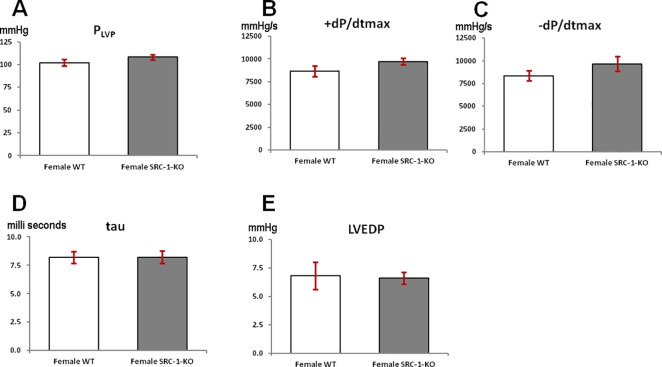
Left ventricular pressure indices. Left ventricular blood pressure parameters of female WT and SRC-1-KO mice. (**A**) Peak left ventricular pressure (P_LVP_), (**B**) Maximal contractiIity (+dP/dt_max_), (**C**) Maximal relaxation (-dP/dt_max_), (**D**) relaxation time constant (tau), and (**E**) left ventricular end diastolic pressure (LVEDP) obtained from the left ventricular pressure of the mice. Data are presented as mean±SEM (n = 5-7/group); *—p*<* 0.05. The information supporting this figure is in S2 Dataset.

Peak left ventricular (P_LVP_), +dP/dt_max_, and −dP/dt_max_ trended higher in female SRC-1-KO mice but were not significantly different when compared to female WT mice (Fig 4A–4C). No differences were observed in tau or LVEDP between the groups (Fig 4D and 4E).

### Echocardiography measurements

**Fig 5:**
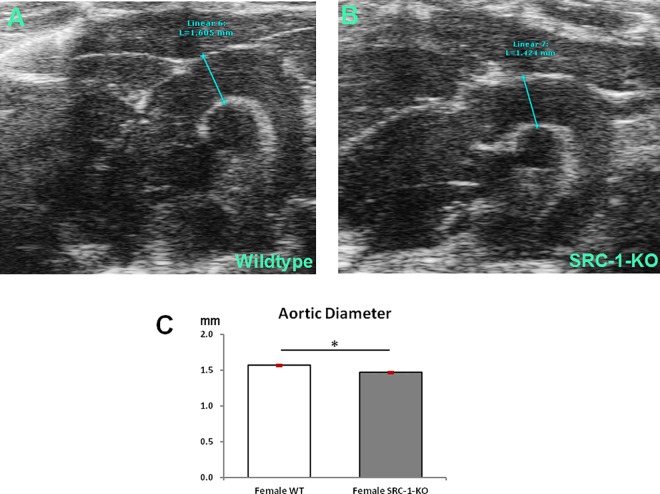
Diameter of the aortic arch. Representative B-mode images of aortic arch in female WT (**A**) and SRC-1-KO (**B**) mice. Quantification (**C**) revealed a decrease in diameter of the aortic arch in SRC-1-KO mice. Data are presented as mean±SEM (n = 4-5/group); *—p*<* 0.05. The information supporting this figure is in S3 Dataset.

Cardiac dimensions and parameters were measured to determine if SRC-1 deletion caused any structural or functional changes in the myocardium. Echocardiography measurements showed no significant differences in any of the parameters between the female SRC-1-KO mice and female WT littermates (Table 1). However, B-Mode imaging of transverse aorta showed that the diameter of the aortic arch in female SRC-1-KO mice was significantly smaller (p = 0.014) than that in female WT littermates (Fig 5A–5C).

**Table 1:** Echocardiographic parameters of female WT and SRC-1 KO mice.

Cardiac	Female	Female
Parameters	WT	SRC-1-KO
**Ds (mm)**	2.12±0.07	1.95±0.06
**Dd (mm)**	3.53±0.08	3.39±0.05
**SV (mm)**	37.5±1.7	35.3±1.4
**EF%**	71.6±0.9	74.5±1.6
**FS%**	40.2±0.9	42.7±1.4
**LVAWd (mm)**	0.71±0.02	0.68±0.03
**LVIDd (mm)**	3.48±0.08	3.49±0.06
**LVPwd (mm)**	0.81±0.03	0.86±0.10
**LVAWs (mm)**	0.85±0.03	0.81±0.03
**LVIDs (mm)**	2.23±0.08	2.07±0.06
**LVPWs (mm)**	1.24±0.02	1.27±0.05
**Heart Rate (bpm)**	493±13	531±17

No significant differences were found in the echocardiographic

parameters between 4 female WT and 6 female SRC-1 KO mice

The information supporting this table is in S6 Dataset.

### Doppler flow velocity measurements

**Fig 6:**
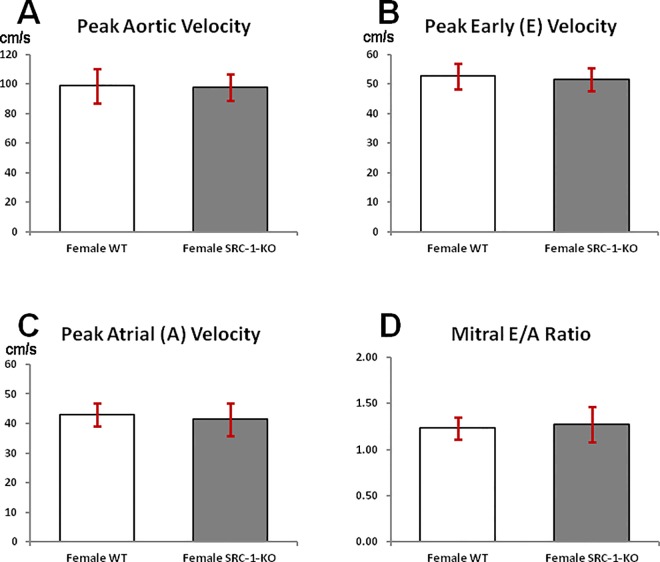
Cardiac flow velocity indices. Peak aortic outflow velocity (**A**), peak mitral-E flow velocity (**B**), peak mitral-A flow velocity (**C**) and mitral E/A ratio (**D**) obtained from cardiac Doppler flow velocity signals in female WT and SRC-1-KO mice. Data are presented as mean±SEM (n = 4-6/group); *—p*<* 0.05. The information supporting this figure is in S4 Dataset.

Assessment of cardiac function using Doppler flow velocity measurements revealed no differences between the genotypes either in the systolic (Fig 6A) or in the diastolic (Fig 6B and 6C) function or in the E/A ratio (Fig 6D). PWV_ff_ was higher in the female SRC-1-KO mice compared to the female WT mice but the differences were not significant (421±17 vs. 388±5 cm/s).

### Aortic input impedance measurements

**Fig 7:**
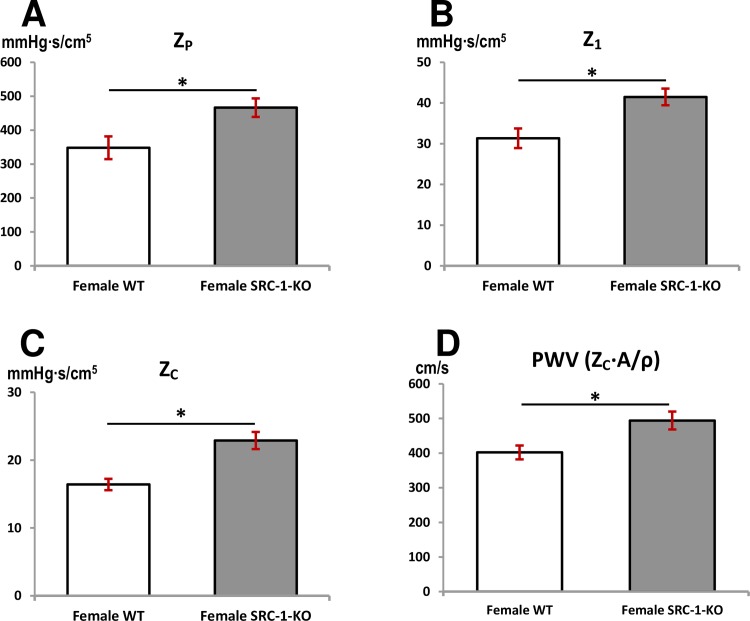
Loss of SRC-1 in female mice results in aortic stiffness. Parameters of aortic impedance in female WT and SRC-1-KO mice. Total peripheral resistance, Z_P_ (**A**), impedance at first harmonic, Z_1_ (**B**), characteristic impedance, Z_C_ (**C**), and impedance based pulse wave velocity, PWV_Z_ (**D**). Data are presented as mean±SEM (n = 4-6/group); * = p*<* 0.05. The information supporting this figure is in S5 Dataset.

Aortic input impedance was determined to study the effect of SRC-1 deletion on the left ventricular afterload. Female SRC-1-KO mice had significantly higher peripheral vascular resistance (Z_P_) compared to female WT littermates (Fig 7A, Z_P_: 466.3±27.4 vs. 348.0±33.4 mmHg∙s/cm^5^, p = 0.028). Impedance at the first harmonic in was significantly higher in female SRC-1-KO mice than in the female WT littermates (Fig 7B, Z_1_: 41.5±2.0 vs. 31.3±2.4 mmHg∙s/cm^5^, p = 0.014). Characteristic impedance (Z_C_), calculated as the average of the 2^nd^ through 10^th^ harmonic, was higher in the female SRC-1-KO mice than the female WT littermates (Fig 7C, Z_C_: 22.9±1.3 vs. 16.4±0.8 mmHg∙s/cm^5^, p = 0.005). Impedance based aortic pulse wave velocity, PWV_Z_, was significantly higher in female SRC-1-KO mice when compared to female WT littermates (Fig 7D).

## Discussion

A significant finding of our studies was that female SRC-1-KO mice had higher mean and diastolic BP compared to female WT littermates. This is similar to the finding that a single nucleotide polymorphism in the SRC-1 gene (rs1550383) resulted in increased BP in women [20]. While the functional relevance of this single nucleotide polymorphism in human SRC-1 gene has not been explored, our observations from SRC-1-KO mice showed direct biological evidence that loss of SRC-1 functions leads to development of hypertension, at least in female animals, similar to the observations made in humans [5]. Additionally, our finding of abundant SRC-1 expression in various regions of the brain in female WT mice was consistent with the previously reported expression pattern in the mouse [27] and the rat brains [33]. Although systolic BP was higher in the SRC-1-KO mice it did not reach the level of significance. It is to be noted that the single nucleotide polymorphism in the SRC-1 gene significantly elevated diastolic blood pressure but did not affect systolic blood pressure [20]. Also, this may be due to the fact that these young female mice are in the early stages of developing hypertension and aortic stiffness. With age, female mice develop hypertension leading to greater induction of aortic stiffness [30]. Another factor may be isoflurane anesthesia, which causes mild arterial vasodilatation resulting in relatively lower peripheral vascular resistance [34] that may lower SBP without necessarily affecting aortic stiffness [35]. The rate pressure product (RPP = SBP x heart rate) which is considered to reflect an indirect measurement of oxygen consumption by the heart [36] was significantly higher in SRC-1-KO mice indicating an increase in the level of energy utilized or workload on the heart, very much similar to the findings of higher RPP in postmenopausal women [36].

Given that estrogen has been long believed to provide anti-hypertensive benefits [5], higher BP in female SRC-1-KO mice also suggests that SRC-1 regulates BP through its interactions with ERs. This is supported by our neuroanatomic observations where we demonstrated that SRC-1 protein is abundantly expressed in multiple regions of the brain that are known to play a role in the regulation of BP. A few of these SRC-1-expressing regions, e.g. the ARC and MeA, also express high levels of ERα [37]. We previously demonstrated that the majority of pro-opiomelanocortin (POMC) neurons within the ARC region co-express SRC-1 [27]. A portion of these POMC neurons also co-express ERα [38] raising the possibility that SRC-1 in POMC neurons may co-activate ERα functions. Central administration of an ERα agonist, namely 1,3,5-tris (4-hydroxyphenyl)-4-propyl-1H-pyrazole (PPT), can enhance the physical interaction between SRC-1 and ERα proteins in mouse hypothalamus, and this interaction is involved in the anti-obesity effects of estrogen in female mice [27]. It is worth noting that POMC neurons have been reported to play a fundamental role in the development of obesity-induced hypertension [39]. Thus, we speculate that the SRC-1-ERα interaction in hypothalamic neurons, including POMC neurons, may be essential for BP control especially in the context of obesity and other metabolic dysfunctions.

Co-existence of SRC-1 and ERα was also apparent in the MeA, a brain region that has been long thought to regulate BP responses to stress in animals [40,41] and humans [42]. Recently, we demonstrated that ERα in MeA neurons is required to mediate vasoprotective effects of estrogen during stress and suggested that this effect of MeA ERα primarily involves the transcription activity of the receptor [14]. Based on these findings, we speculate that SRC-1 in the MeA may co-activate ERα transcription activity to regulate BP in response to stress.

Abundant SRC-1 was also observed in the PVN region. Although ERα expression is scarce in the PVN, ERβ is highly expressed in this region [37]. While, pharmacological activation of ERβ in the PVN has been reported to attenuate glutamate-induced hypertensive responses in rats [43], siRNA-mediated knockdown of ERβ selectively in the PVN potentiates aldosterone-induced hypertension in mice [44].

While this neuroanatomic evidence provides a basis that SRC-1 may regulate BP through its interactions with ERs in the brain, future investigations are needed to further support these possibilities. Additionally, we could not rule out that SRC-1 may regulate BP via ER-independent mechanisms given the large effects of SRC-1 to co-activate other nuclear receptors. It is worth noting that SRC-1 and SRC-3 (another member of SRC family) are transiently expressed by cardiomyocytes during the perinatal period and play essential roles in cardiomyocyte proliferation and differentiation at earlier developmental stages [45]. Therefore, in addition to the brain, SRC-1 may also act in the peripheral tissues (e.g. the heart and blood vessels) to regulate BP.

Comparison of cardiac dimensions and function showed no significant differences between female SRC-1-KO mice and their WT littermates, suggesting that the loading conditions may be in the initial stages of hypertrophy (due to young age) and the heart may be experiencing physiological hypertrophy rather than pathological hypertrophy. Additionally, no significant differences were observed in either cardiac blood flow velocities between the genotypes indicating that neither the systolic nor the diastolic function is significantly altered.

Aortic impedance, defined as the ratio of pressure to velocity (ratio of frequency components), provides a more complete description of the afterload experienced by the left ventricle by describing the pulsatile and the steady components of the hydraulic load [30,46]. Impedance at zero frequency (Z_P_) is known as total peripheral vascular resistance and is equal to ratio of mean pressure to mean velocity or mean flow. Since mean velocity was not different between the groups, the increase in Z_P_ could be attributed to higher mean pressure in the SRC-1-KO mice. Waddell et al. reported that MAP was higher in older women when compared to young women [6]. Given the young age of the mice in this study, the female WT mice may mimic the young women and the female SRC-1-KO may mimic older (menopausal) women. Since estrogen plays a role in the defense against angiotension-II induced hypertension, we would expect that lack of estrogen function associated with SRC-1 deficiency would cause blood pressure and peripheral vascular resistance to increase. Impedance at the first harmonic (Z_1_) (at heart rate in Hz or beats/second) represents the strength of the wave reflections from the periphery. Significant elevation of Z_1_ in female SRC-1-KO may allude to a greater mismatch between ventriculo-vascular coupling [47]. While higher peripheral resistance causes stronger wave reflections, stiffer aorta will cause these reflections to arrive early in cardiac cycle worsening the mismatch of the ventricular-arterial coupling [48]. Higher characteristic impedance (Z_C_) and PWV_Z_, influenced by aortic diameter and the viscoelastic properties of the aortic wall [49], indicate a stiffer aorta in the female SRC-1-KO mice, very much similar to that reported in older (post-menopausal ages) women [50]. Higher blood pressures did not increase aortic PWV_ff_ in female SRC-1-KO mice which may be due to the fact that PWV_ff_ was measured at end diastolic pressure where the aorta is not as distended and the effects of stiffness are not as pronounced as would be the case at peak systolic pressure [29]. Comparatively, PWV_Z_ was reflective of higher blood pressure and a better indicator of aortic stiffness as it represents the average PWV in the entire cardiac cycle unlike PWV_ff_ which is measured only at end diastolic pressure[29,51].

### Limitations of the study

While some of the parameters showed significant phenotype differences, others parameters either showed subtle to no differences. Despite genetic differences, the phenotype differences between transgenic animals and their wild type counter parts tend to be masked at baseline. We did not conduct any interventional studies that may have had the potential to bring forth the phenotypic differences in these animals. Additionally, the studies were done under isoflurane anesthesia which is known to mildly depress blood pressure and may also mask phenotypic differences. Future studies will include interventions to better delineate the phenotypic differences, specifically when measurements are made under anesthesia.

### Conclusions

In conclusion, we demonstrated that SRC-1 is expressed in hypothalamic (PVN, SON, ARC, DMH, and VMH), extra hypothalamic (CeA, MeA), and brain stem (NTS) neuronal sites known to regulate BP. Furthermore, we found that deletion of SRC-1 leads to increased BP in female mice and higher aortic input impedance. Additionally, we found that female SRC-1-KO mice had decreased aorta diameter and higher PWV_Z_, indicating that they may have stiffer aorta. Together, our results demonstrate that SRC-1 is functionally required for protection against hypertension in females.

## Supporting Information

S1 Dataset Aortic blood pressure indices, heart rate, and rate pressure product. (PDF)

S2 Dataset Left ventricular pressure indices and maximal rates of contraction and relaxation. (PDF)

S3 Dataset Aortic arch diameters. (PDF)

S4 Dataset Doppler flow velocity indices in the heart. (PDF)

S5 Dataset Aortic impedance indices. (PDF)

S6 Dataset Echocardiographic indices. (PDF)
