# Supplementary material for: SRC-1 Regulates Blood Pressure and Aortic Stiffness in Female Mice
Source: PLoS One. 2016 Dec 22;11(12):e0168644. doi: 10.1371/journal.pone.0168644 (PMC5179266; doi:10.1371/journal.pone.0168644)
Supplement: S1 Dataset — (PDF) [file pone.0168644.s001.pdf]

SRC-1 Regulates Blood Pressure and Aortic Stiffness in Female Mice

Antentor Othrell Hinton Jr., Yongjie Yang, Ann P. Quick, Pingwen Xu, Chitra L. Reddy, Xiaofeng Yan, Corey L. Reynolds, Qingchun Tong, Liangru Zhu, Jianming Xu, Xander H. T. Wehrens, Yong Xu, Anilkumar K. Reddy

Supporting Information

S1 Dataset. Aortic blood pressure indices, heart rate, and rate pressure product. Individual data samples of systolic blood pressure, diastolic blood pressure, mean blood pressure, pulse pressure, heart rate, and rate pressure product (systolic blood pressure x heart rate) of female WT and SRC-1-KO mice (dataset for Figure 3).

Mouse Genotype	Systolic BP (mmHg)	Diastolic BP (mmHg)	Mean BP (mmHg)	Pulse Pressure (mmHg)	Heart Rate (bpm)	Rate x Systolic BP (bpm x mmHg)
**WT1**	**102.7**	**71.7**	**86.5**	**31.0**	**451**	**46318**
**WT2**	**98.8**	**65.8**	**81.5**	**33.1**	**448**	**44271**
**WT3**	**104.9**	**71.9**	**86.9**	**33.0**	**461**	**48340**
**WT4**	**114.2**	**75.7**	**93.4**	**38.4**	**451**	**51483**
**SRC-1 KO1**	**112.2**	**74.8**	**90.7**	**37.4**	**481**	**53944**
**SRC-1 KO2**	**119.7**	**79.9**	**99.2**	**39.8**	**507**	**60693**
**SRC-1 KO3**	**111.2**	**77.1**	**93.2**	**34.1**	**484**	**53806**
**SRC-1 KO4**	**114.7**	**77.2**	**94.9**	**37.5**	**444**	**50916**
**SRC-1 KO5**	**105.3**	**74.8**	**88.9**	**30.6**	**473**	**49811**
**SRC-1 KO6**	**113.7**	**79.4**	**95.3**	**34.3**	**537**	**61057**
