# Supplementary material for: SRC-1 Regulates Blood Pressure and Aortic Stiffness in Female Mice
Source: PLoS One. 2016 Dec 22;11(12):e0168644. doi: 10.1371/journal.pone.0168644 (PMC5179266; doi:10.1371/journal.pone.0168644)
Supplement: S2 Dataset — (PDF) [file pone.0168644.s002.pdf]

SRC-1 Regulates Blood Pressure and Aortic Stiffness in Female Mice

Antentor Othrell Hinton Jr., Yongjie Yang, Ann P. Quick, Pingwen Xu, Chitra L. Reddy, Xiaofeng Yan, Corey L. Reynolds, Qingchun Tong, Liangru Zhu, Jianming Xu, Xander H. T. Wehrens, Yong Xu, Anilkumar K. Reddy

Supporting Information

S2 Dataset. Left ventricular pressure indices and maximal rates of contraction and relaxation. Individual data samples of peak left ventricular pressure, maximal +dP/dt, maximal -dP/dt, relaxation time constant (tau), and left ventricular end diastolic pressure (LVEDP) of female WT and SRC-1-KO mice (dataset for Figure 4).

Mouse Genotype	Peak LV Pressure (mmHg)	+dP/dtmax (mmHg/s)	-dP/dtmax (mmHg/s)	tau (ms)	LVEDP (mmHg)
WT1	101.8	7875	7914	8.65	6.8
WT2	101.0	9861	8675	6.76	4.8
WT3	93.3	7102	6871	9.21	10.8
WT4	112.4	9788	9948	7.26	4.9
SRC-1 KO1	116.6	11063	10821	5.62	5.8
SRC-1 KO2	106.8	8246	8724	10.44	6.7
SRC-1 KO3	112.2	11536	10885	7.03	3.7
SRC-1 KO4	116.4	9385	8731	9.07	5.8
SRC-1 KO5	98.9	8965	7874	8.73	8.5
SRC-1 KO6	109.4	10852	12376	6.11	5.5
