# Supplementary material for: SRC-1 Regulates Blood Pressure and Aortic Stiffness in Female Mice
Source: PLoS One. 2016 Dec 22;11(12):e0168644. doi: 10.1371/journal.pone.0168644 (PMC5179266; doi:10.1371/journal.pone.0168644)
Supplement: S3 Dataset — (PDF) [file pone.0168644.s003.pdf]

SRC-1 Regulates Blood Pressure and Aortic Stiffness in Female Mice

Antentor Othrell Hinton Jr., Yongjie Yang, Ann P. Quick, Pingwen Xu, Chitra L. Reddy, Xiaofeng Yan, Corey L. Reynolds, Qingchun Tong, Liangru Zhu, Jianming Xu, Xander H. T. Wehrens, Yong Xu, Anilkumar K. Reddy

Supporting Information

S3 Dataset. Aortic arch diameters. Individual data samples of aortic arch diameter of female WT and SRC-1-KO mice (dataset for Figure 5).

Mouse Genotype	Aortic Diameter (mm)
**WT1**	**1.551**
**WT2**	**1.568**
**WT3**	**1.605**
**SRC-1 KO1**	**1.494**
**SRC-1 KO2**	**1.477**
**SRC-1 KO3**	**1.424**
