# Supplementary material for: SRC-1 Regulates Blood Pressure and Aortic Stiffness in Female Mice
Source: PLoS One. 2016 Dec 22;11(12):e0168644. doi: 10.1371/journal.pone.0168644 (PMC5179266; doi:10.1371/journal.pone.0168644)
Supplement: S4 Dataset — (PDF) [file pone.0168644.s004.pdf]

SRC-1 Regulates Blood Pressure and Aortic Stiffness in Female Mice

Antentor Othrell Hinton Jr., Yongjie Yang, Ann P. Quick, Pingwen Xu, Chitra L. Reddy, Xiaofeng Yan, Corey L. Reynolds, Qingchun Tong, Liangru Zhu, Jianming Xu, Xander H. T. Wehrens, Yong Xu, Anilkumar K. Reddy

Supporting Information

S4 Dataset. Doppler flow velocity indices in the heart. Individual data samples of peak aortic flow velocity, peak mitral early (E) flow velocity, peak atrial (A) flow velocity, and E/A peak flow velocity ratio of female WT and SRC-1-KO mice (dataset for Figure 6).

Mouse Genotype	Pk Aortic Vel. (cm/s)	Pk E-Vel. (cm/s)	Pk A-Vel. (cm/s)	E/A Ratio
**WT1**	**91.7**	**51.6**	**38.5**	**1.3**
**WT2**	**117.7**	**58.5**	**42.6**	**1.4**
**WT3**	**87.1**	**46.6**	**41.5**	**1.1**
**WT4**	**98.5**	**53.8**	**49.1**	**1.1**
**SRC-1 KO1**	**84.3**	**55.5**	**43.2**	**1.3**
**SRC-1 KO2**	**106.8**	**53.8**	**33.4**	**1.6**
**SRC-1 KO3**	**94.4**	**49.5**	**47.1**	**1.1**
**SRC-1 KO4**	**108.1**	**47.1**	**34.2**	**1.4**
**SRC-1 KO5**	**90.0**	**56.7**	**45.9**	**1.2**
**SRC-1 KO6**	**102.8**	**46.9**	**44.4**	**1.1**
