# Supplementary material for: SRC-1 Regulates Blood Pressure and Aortic Stiffness in Female Mice
Source: PLoS One. 2016 Dec 22;11(12):e0168644. doi: 10.1371/journal.pone.0168644 (PMC5179266; doi:10.1371/journal.pone.0168644)
Supplement: S5 Dataset — (PDF) [file pone.0168644.s005.pdf]

SRC-1 Regulates Blood Pressure and Aortic Stiffness in Female Mice

Antentor Othrell Hinton Jr., Yongjie Yang, Ann P. Quick, Pingwen Xu, Chitra L. Reddy, Xiaofeng Yan, Corey L. Reynolds, Qingchun Tong, Liangru Zhu, Jianming Xu, Xander H. T. Wehrens, Yong Xu, Anilkumar K. Reddy

Supporting Information

S5 Dataset. Aortic impedance indices. Individual data samples of peripheral vascular resistance (Z_P), impedance at first harmonic (Z_1), characteristic impedance (Z_C), impedance based pulse wave velocity (PWV_Z), and foot-to-foot based pulse wave velocity (PWV_{ff}) of female WT and SRC-1-KO mice (dataset for Figure 7).

Mouse Genotype	Z_P ($mmHg \cdot s/cm^5$)	Z_1 ($mmHg \cdot s/cm^5$)	Z_C ($mmHg \cdot s/cm^5$)	PWV_Z cm/s	PWV_{ff} cm/s
**WT1**	**367**	**30.1**	**18.2**	**446**	**391**
**WT2**	**249**	**25.2**	**14.2**	**348**	**380**
**WT3**	**387**	**33.7**	**17.0**	**417**	**381**
**WT4**	**389**	**36.3**	**16.2**	**397**	**400**
**SRC-1 KO1**	**526**	**48.4**	**25.4**	**538**	**469**
**SRC-1 KO2**	**364**	**36.6**	**23.6**	**500**	**389**
**SRC-1 KO3**	**476**	**40.7**	**21.1**	**447**	**398**
**SRC-1 KO4**	**495**	**38.8**	**19.9**	**422**	**370**
**SRC-1 KO5**	**470**	**43.0**	**26.5**	**562**	**443**
**SRC-1 KO6**	**-**	**-**	**-**	**-**	**459**
