# Supplementary material for: SRC-1 Regulates Blood Pressure and Aortic Stiffness in Female Mice
Source: PLoS One. 2016 Dec 22;11(12):e0168644. doi: 10.1371/journal.pone.0168644 (PMC5179266; doi:10.1371/journal.pone.0168644)
Supplement: S6 Dataset — (PDF) [file pone.0168644.s006.pdf]

SRC-1 Regulates Blood Pressure and Aortic Stiffness in Female Mice

Antentor Othrell Hinton Jr., Yongjie Yang, Ann P. Quick, Pingwen Xu, Chitra L. Reddy, Xiaofeng Yan, Corey L. Reynolds, Qingchun Tong, Liangru Zhu, Jianming Xu, Xander H. T. Wehrens, Yong Xu, Anilkumar K. Reddy

Supporting Information

S6 Dataset. Echocardiographic indices. Individual data samples of left ventricular internal diameter-systole (Ds), left ventricular internal diameter-diastole (Dd), stroke volume (SV), ejection fraction (EF), fractional shortening (FS), left ventricular anterior wall thickness-diastole (LVAWd), left ventricular internal diameter-diastole (LVIDd), left ventricular posterior wall thickness-diastole (LVPWd), left ventricular anterior wall thickness-systole (LVAWs), left ventricular internal diameter-systole (LVIDs), left ventricular posterior wall thickness-systole (LVPWs), and heart rate of female WT and SRC-1-KO mice (dataset for Table 1).

Mouse Genotype	Ds (mm)	Dd (mm)	SV (µl)	EF (%)	FS (%)	LVAWd (mm)	LVIDd (mm)	LVPwd (mm)	LVAWs (mm)	LVIDs (mm)	LVPWs (mm)	Echo Heart Rate (bpm)
**WT1**	**1.94**	**3.40**	**35.6**	**74.5**	**42.8**	**0.642**	**3.35**	**0.795**	**0.783**	**2.06**	**1.30**	**534**
**WT2**	**2.33**	**3.78**	**42.2**	**69.3**	**38.3**	**0.752**	**3.72**	**0.793**	**0.853**	**2.47**	**1.18**	**461**
**WT3**	**2.14**	**3.59**	**38.9**	**71.9**	**40.5**	**0.697**	**3.53**	**0.794**	**0.818**	**2.26**	**1.24**	**497**
**WT4**	**2.06**	**3.38**	**33.1**	**70.7**	**39.1**	**0.760**	**3.31**	**0.870**	**0.940**	**2.13**	**1.23**	**482**
**SRC-1 KO1**	**2.18**	**3.42**	**32.3**	**67.1**	**36.3**	**0.559**	**3.41**	**0.740**	**0.680**	**2.27**	**1.06**	**482**
**SRC-1 KO2**	**1.95**	**3.30**	**32.4**	**72.2**	**41.1**	**0.620**	**3.20**	**0.846**	**0.733**	**2.05**	**1.23**	**575**
**SRC-1 KO3**	**1.94**	**3.49**	**38.9**	**76.8**	**44.5**	**0.664**	**3.51**	**0.744**	**0.833**	**2.07**	**1.36**	**493**
**SRC-1 KO4**	**1.69**	**3.20**	**32.9**	**79.9**	**47.3**	**0.752**	**3.15**	**0.870**	**0.831**	**1.83**	**1.35**	**524**
**SRC-1 KO5**	**1.86**	**3.32**	**34.4**	**76.3**	**44.0**	**0.746**	**3.26**	**0.870**	**0.879**	**1.97**	**1.42**	**600**
**SRC-1 KO6**	**2.07**	**3.61**	**41.0**	**74.7**	**42.7**	**0.720**	**3.57**	**1.050**	**0.875**	**2.25**	**1.21**	**512**
